# The effect of blood flow restriction training on core muscle strength and pain in male collegiate athletes with chronic non-specific low back pain

**DOI:** 10.3389/fpubh.2024.1496482

**Published:** 2025-01-07

**Authors:** Yixuan Liu, Jiahuan Liu, Min Liu, Minzhuo Wang

**Affiliations:** ^1^Institute of Physical Education, Shanxi University, Taiyuan, China; ^2^School of Marxism, Central University of Finance and Economics, Beijing, China

**Keywords:** blood flow restriction training (BFRT), resistance training (RT), chronic non-specific low back pain, training strategy, rehabilitation

## Abstract

**Objective:**

The objective of this study is to compare the effectiveness of low-load blood flow restriction training (LL-BFRT) to heavy-load resistance training (HL-RT) in male collegiate athletes with chronic non-specific low back pain (CNLBP).

**Methods:**

Twenty-six participants were randomly assigned to LL-BFRT (*n* = 13) or HL-RT (*n* = 13). All participants supervised exercises (deep-squat, lateral pull-down, bench-press and machine seated crunch) cycled 4 times per week for 4 weeks (16 sessions). LL-BFRT was done at 30% 1-repetition maximum (1RM) with 70% arterial occlusion pressure (AOP). HL-RT was done at 70% 1-RM. The outcomes were isokinetic core strength, isometric core endurance, pain intensity, and lumbar function disability level, measured at baseline and 4 weeks. Intra-group differences were evaluated using *t*-tests.

**Results:**

Pain intensity and function disability level in LL-BFRT had extremely significant improvement at 4 weeks (*p* < 0.001, ES = 1.44–1.84). Participants in LL-BFRT and HL-RT showed significant differences in core extensors peak torque-body weight ratio (PT/BW) at isokinetic 120°/s and 30°/s, respectively (LL-BFRT: *p* = 0.045, ES = 0.62; HL-RT: *p* = 0.013, ES = 0.81). Isometric core extensor endurance was significantly increased in both groups (LL-BFRT: *p* = 0.016, ES = 0.78; HL-RT: *p* = 0.011, ES = 0.83).

**Conclusion:**

Four weeks of LL-BFRT significantly reduced pain and functional disability while inducing similar strength gains as HL-RT in male collegiate athletes with CNLBP. Thereby, BFRT may qualify as a valuable training strategy for people with physical limitations.

## 1 Introduction

Chronic low back pain (CLBP, pain lasting more than 12 weeks duration) is one of the most common chronic musculoskeletal disorders, and it is a widespread public health concern because of its high prevalence rates worldwide (1, 2). Patients with CLBP have varying degrees of pain in the lumbar region of the spine, generally located between the lower ribs and the gluteal region (3). Approximately 85% of patients have chronic non-specific low back pain (CNLBP) who do not have a specific patho-anatomical cause attributable to their pain in clinical examination (4). CNLBP is the most common musculoskeletal condition impacting athletes' performance and involvement in sports (5). Atrophy and fatty infiltration in the lumbar multifidus and transverse abdominal muscles are the main causes of CNLBP, and exercise therapy aimed at recovering activation and endurance of these muscles enhances the biomechanical mechanisms of CNLBP patients (6).

In view of the effectiveness of strength training in CNLBP, current research is centered on optimizing training methods in order to enhance its effects further. The American College of Sports Medicine (ACSM) suggests that significant adaptation to resistance exercise requires at least 70% of 1RM to enhance strength (7). However, it has been demonstrated that a number of patients with musculoskeletal pain may have difficulties bearing the training loads required to achieve the clinical benefits of rehabilitation training. The core muscle strength impairment leads to low lumbar spine stability in patients with CNLBP. High-load training can exacerbate the patient's muscle imbalance and the biomechanical structure of the spine, increasing the risk of sports injuries (1, 8).

In conclusion, muscle strength impairment remains a persisting problem in CNLBP, reinforcing the vicious circle of pain and trunk muscle imbalance. An underlying reason might be the unavailability of bearable gain-inducing strength training loads.

Blood flow restriction training (BFRT) is a method to increase muscle strength with low loads, whereby arterial blood flow to the trained limb is restricted by the inflation of an air cuff (9). LL-BFRT is usually performed at 20%–40% of the 1-RM, which could achieve comparable muscle mass and strength gains to HL-RT (10–12). In addition, evidence supports that LL-BFRT can reduce pain significantly while improving muscle strength in patients with musculoskeletal disorders (13).

To our knowledge, the potential effectiveness of LL-BFRT in patients with CNLBP has not been discussed and explored. Thus, the goal of the randomized controlled trial was to compare the effect of LL-BFRT to HL-RT in pain intensity, core strength, and self-rated improvement of low back function in male collegiate athletes with CNLBP. We hypothesized that LL-BFRT would reduce pain and improve low back function while increasing core muscle strength with the lower training loads.

## 2 Materials and methods

### 2.1 Participants

This study openly recruited individuals aged 18–24 years with CNLBP lasting more than 12 weeks. Twenty-six male collegiate athletes with CNLBP volunteered to participate in the study and had a training period of 3–6 years. They were divided into LL-BFRT (*n* = 13) and HL-RT (*n* = 13) by the random number table method. The participants of LL-BFRT mean (±SD) age, height, and weight were 21.23 ± 2.13 years, 181.22 ± 6.61 cm, and 82.51 ± 13.28 kg, respectively. In HL-RT, the participants' mean (±SD) age, height, and weight were 21.23 ± 2.17 years, 184.53 ± 4.75 cm, and 84.48 ± 12.36 kg.

Prior to testing participants provided signed informed consent after the nature and goals of the study had been thoroughly explained. The study was approved by the Shanxi University Ethics Committee (No. SXDXLL2024102). CNLBP in this study was defined as follows (14): (1) persistent pain localized below the costal margin and above the inferior gluteal folds for more than 12 weeks; (2) the absence of specific spinal pathologies such as infection, tumors, and vertebral fractures on both plain radiographs and lumbar magnetic resonance imaging (MRI); (3) the absence of dominant leg pain caused by radicular and cauda equina disorders; (4) the absence of prominent instability such as spondylolysis, isthmic spondylolisthesis, and degenerative spondylolisthesis more than grade II; and (5) no previous lumbar and/or thoracolumbar spine surgery. Degenerated lumbar structures such as the vertebral disc, facet joint, and sacroiliac joint were omitted from the inclusion criteria. Exclusion criteria included: (1) individuals who did not cooperate with training after inclusion; (2) cases of other injuries during the trial; (3) Cases of adverse events occurring during the trial; and (4) Cases of voluntary withdrawal during the trial.

### 2.2 Methods

#### 2.2.1 Experimental design

This study was a randomized controlled trial. Participants initially completed a series of baseline tests and became familiar with the training used in the study. All participants performed an isometric core endurance test and isokinetic core strength test and completed VAS and ODI questionnaires (15–18). Participants completed low-load resistance training with blood flow restriction or heavy-load resistance training before repeating the test protocols. The training consisted of 4 weekly exercise sessions for 4 weeks, totaling 16 sessions. The test content, researchers, and test instruments were the same. The participants were instructed to avoid other physical training or therapy during the 4 weeks of the training. Baseline and follow-up tests were conducted at 24 h before and after formal training.

#### 2.2.2 Training protocol

There were 4 sessions per week (Mondays, Wednesdays, Fridays, and Sundays), all held in the morning. Each session included deep-squat, lateral pull-down, bench-press, and machine seated crunch. In order to determine the training load, all participants had finished 1 RM test 48 h before the 4 weeks of training. Participants were divided into two groups to perform resistance exercises. In the LL-BFRT group, participants performed blood flow restriction training at 30% 1RM. Each exercise consisted of 4 sets. The first set was repeated 30 times, and the others were repeated 15 times. The rest intervals were standardized to 60s (19). Participants in LL-BFRT placed pressure cuffs proximally on both arms or thighs receiving BFR and inflated to the pressure of 70%. The cuff pressure was sustained throughout the exercise but was released between sets. The HL-RT participants performed 70% 1RM resistance training for 4 sets of 15 repetitions with an interval of 90s. Components of the training protocol are presented in Table 1 for both groups.

**Table 1:** Components of the training protocol.

		**LL-BFRT group**	**HL-RT group**
Frequency		Four sessions per week for 4weeks, Mondays, Wednesdays, Fridays, and Sundays	
Exercises		Deep-squat, lateral pull-down, bench-press, and machine seated crunch	
Intensity			
Volume		4 sets (30-15-15-15 repetitions)	4 sets (15 repetitions)
Load		30% of 1RM	70% of 1RM
Rest		60s between sets	90s between sets

1-RM, 1-repetition maximum.

#### 2.2.3 BFR

Following a 5-min light jog warm-up, an 8 cm wide inflatable cuff (BSTRONG Blood Flow Restriction Training Kit, BStrong USA) was secured and inflated at the most proximal part of arms or thighs (20). The inflation pressure was set at 180 mm Hg. LL-BFRT was done at 30% 1-RM with an arterial occlusion pressure (AOP) of 70%, in accordance with available evidence-based application guidelines (21). The air valve was then tightened to maintain the target pressure.

### 2.3 Outcome measures

#### 2.3.1 Pain intensity and functional disability level assessment

The Visual Analog Scale (VAS) effectively assesses participants' low back pain (16). It is a simple, effective, and repeatable tool providing a rapid measurement of pain severity in clinical and laboratory conditions. The patient was asked to mark a place on a 10 cm horizontal line that showed his current status, with 0 indicating no pain and 10 indicating severe pain (17).

The Oswestry Disability Index (ODI) is a valid and reliable tool for assessing disability due to lower back pain (16). It consisted of 10 items across three domains (pain, single-item function, and overall function) and was self-administered to assess the limitations of different activities of daily living. Each item is scored on a scale of 5 points, with a total possible score of 50 points. A score of 0 indicates no functional impairment, with higher scores indicating higher disability (22).

#### 2.3.2 Isometric core endurance test

Core endurance was evaluated using the McGill endurance test, which is considered the most reliable isometric test for evaluating core muscle endurance and stability (23). Trunk extensors endurance test: The starting position required the participant to be prone, positioning the iliac crests at the table edge while supporting the upper extremity on the arms, which were placed on the floor or a riser. While the participant was supporting the weight of his upper body, he anchored the participant's lower legs to the table using a strap. When ready, the participant lifted and extended the torso until it was parallel to the floor, with the upper limbs held across the chest and the hands resting on the opposite shoulders. The participant was instructed to keep a horizontal, prone position for as long as possible. Failure occurred when the upper body dropped below the horizontal position.

Trunk flexors endurance test: The test began with the participant sitting up with the back resting against a jig angled at 60 degrees from the floor. Both knees and hips were flexed 90 degrees, the arms were folded across the chest, and the feet were secured. The jig was pulled back 10 cm, and the participant held the isometric posture as long as possible. The test is terminated once any part of the participant's back touches the jig.

The stopwatch was started as soon as the participant assumed the starting position, and the test should be terminated when participants can no longer maintain the position. The researcher accurately recorded the duration of the hold.

#### 2.3.3 Isokinetic core strength test

Torque and angular velocity data were collected using the German-manufactured IsoMed2000. It has been confirmed in the past that fixation at the anterior superior iliac spine results in correct and reliable measurements of the core musculature in standing position (15). With participants standing, fixation is performed at the shoulders, pelvis, and knees. The knee joint was flexed to approximately 15 degrees. The testing mode was set to concentric-concentric. Lumbar flexion and extension measurements are performed in isokinetic 30°/s, isokinetic 90°/s, and isokinetic 120°/s for 5 reputations with a 30-second rest period between sets (24). A 10-min rest period was carried out between testing sessions at different angular velocities to reduce the impact of fatigue to a minimum.

This study collected each patient's peak torque-body weight ratio (PT/BW) of the lumbar flexor and extensor. PT/BW provides a better indication of the relative strength of the muscle because it eliminates the influence of body weight factors. The flexion-extension ratio (E/F) was also determined in the test. E/F offers valuable information about the balance of muscle strength around the lumbar.

#### 2.3.4 Statistical analysis

For a clinical trial study, the recommendation is a sample size of 12 per group, owing to the rationale about feasibility and precision of the mean and variance (25). We aimed to include 26 participants, considering a drop-out rate of 10% (i.e., 13/group).

All data were screened for normal distribution using the Shapiro–Wilk test. To compare variables among baseline and 4 weeks, a paired samples *T*-test was performed. An independent-sample *T*-test was employed to calculate the change rates of various indicators. When the data was in a normal distribution, the 95% confidence interval (CI) for the difference in means was computed. Cohen's d was used to describe the group effect sizes, calculated as the difference in the means divided by the pooled SD. The effect size was regarded as small when 0.2 ≤ d < 0.5, medium when 0.5 ≤ d < 0.8, and large when d ≤ 0.8 (26). All data were analyzed with IBM SPSS, and the level of significance was set at 0.05. Descriptive data are presented as the mean ± SD.

## 3 Results

### 3.1 VAS and ODI

The LL-BFRT group was statistically superior to the HL-RT group in VAS and ODI (Table 2). VAS and ODI increased extremely significantly in the LL-BFRT group (*p* < 0.001), while they increased significantly in the HL-RT group (*p* < 0.05).

**Table 2:** Changes in VAS and ODI scores across study groups.

	**VAS score**		**t**	***p* value**	***ES***	**ODI score**		**t**	***p* value**	***ES***
	**Pre-training**	**Post-training**				**Pre-training**	**Post-training**			
LL-BFRT	5.54 ± 1.39	3.92 ± 1.38^***^	5.196	0.001	1.44	28.85 ± 6.82	21.62 ± 4.94^***^	6.617	0.001	1.84
HL-RT	5.46 ± 1.66	4.62 ± 1.33^*^	2.513	0.027	0.7	29.38 ± 7.90	27.38 ± 6.85^*^	2.576	0.024	0.71

Values are presented as means ± SD. ^*^Significantly different from pre-training (*p* < 0.05). ^***^Extremely significantly different from pre-training (*p* < 0.001). LL-BFRT, low-load blood flow restriction training; HL-RT, heavy-load resistance training; VAS, Visual Analog Scale; ODI, Oswestry Disability Index.

### 3.2 Isometric core endurance

After the 4-week training, the endurance duration of the extensors in both groups showed a significant increase from pre-training levels (*p* < 0.05; Table 3), whereas no statistically significant difference was observed in flexors endurance duration (*p* > 0.05).

**Table 3:** Changes in isometric core endurance of the flexors and extensors across study groups.

		**Core static endurance/s**		**t**	***p* value**	***ES***
		**Pre-training**	**Post-training**			
Extensors	LL-BFRT	129.95 ± 18.23	138.28 ± 17.11^*^	−2.818	0.016	0.78
	HL-RT	126.54 ± 18.39	139.01 ± 20.75^*^	−2.997	0.011	0.83
Flexors	LL-BFRT	100.13 ± 17.64	103.50 ± 18.96	−0.955	0.358	0.32
	HL-RT	100.14 ± 17.15	105.01 ± 20.97	−1.896	0.082	0.53

Values are presented as means ± SD. ^*^ Significantly different from pre-training (*p* < 0.05). LL-BFRT, low-load blood flow restriction training; HL-RT, heavy-load resistance training.

### 3.3 Isokinetic core strength

The results of the PT/BW are presented in Table 4. After the 4-week training, in terms of extensors, the PT/BW in the HL-RT group showed a very significant increase at 30°/s (*p* < 0.05). However, the PT/BW substantially increased at 120°/s in the LL-BFRT group (*p* < 0.05). The difference was insignificant in flexors PT/BW at any speed (*p* > 0.05).

**Table 4:** Changes in isokinetic trunk extension and flexion PT/BW across study groups.

			**PT/BW**		**t**	***p* value**	***ES***
			**Pre-training**	**Post-training**			
30°/s	Extensors	LL-BFRT	4.56 ± 0.70	4.63 ± 0.77	−1.695	0.116	0.47
		HL-RT	4.58 ± 0.65	4.72 ± 0.67^*^	−2.922	0.013	0.81
	Flexors	LL-BFRT	4.13 ± 0.67	4.18 ± 0.72	−0.956	0.358	0.27
		HL-RT	4.15 ± 0.54	4.20 ± 0.57	−0.994	0.34	0.28
90°/s	Extensors	LL-BFRT	4.12 ± 0.63	4.20 ± 0.58	−1.746	0.106	0.48
		HL-RT	4.21 ± 0.67	4.28 ± 0.69	−0.896	0.388	0.25
	Flexors	LL-BFRT	4.05 ± 0.61	4.08 ± 0.56	−0.643	0.532	0.18
		HL-RT	4.07 ± 0.55	4.11 ± 0.56	−0.671	0.515	0.19
120°/s	Extensors	LL-BFRT	3.84 ± 0.71	3.98 ± 0.71^*^	−2.241	0.045	0.62
		HL-RT	3.90 ± 0.63	3.97 ± 0.61	−1.330	0.208	0.37
	Flexors	LL-BFRT	4.02 ± 0.63	4.06 ± 0.67	−1.652	0.124	0.45
		HL-RT	4.03 ± 0.56	4.05 ± 0.57	−1.442	0.175	0.40

Values are presented as means ± SD. ^*^ Significantly different from pre-training (*p* < 0.05). LL-BFRT, low-load blood flow restriction training; HL-RT, heavy-load resistance training; PT/BW, peak torque-body weight ratio.

Table 5 exhibits the flexion-extension ratio of isokinetic trunk peak torque before and after 4 weeks of training. When the angular velocity was 30°/s, there were significant differences in the HL-RT group (*p* < 0.05), and there were significant changes in the LL-BFRT group at 120°/s (*p* < 0.05).

**Table 5:** Changes in F/E of isokinetic trunk peak torque across study groups.

		**F/E**		**t**	***p* value**	***ES***
		**Pre-training**	**Post-training**			
30°/s	LL-BFRT	0.91 ± 0.03	0.90 ± 0.06	0.216	0.833	0.06
	HL-RT	0.91 ± 0.04	0.89 ± 0.04^*^	2.416	0.033	0.67
90°/s	LL-BFRT	0.98 ± 0.05	0.97 ± 0.04	1.614	0.132	0.45
	HL-RT	0.97 ± 0.06	0.97 ± 0.06	0.757	0.464	0.21
120°/s	LL-BFRT	1.04 ± 0.09	1.02 ± 0.09^*^	2.186	0.049	0.37
	HL-RT	1.04 ± 0.07	1.02 ± 0.07	1.272	0.227	0.35

Values are presented as means ± SD. ^*^Significantly different from pre-training (*p* < 0.05). LL-BFRT, low-load blood flow restriction training; HL-RT, heavy-load resistance training; F/E, flexion-extension ratio.

## 4 Discussion

### 4.1 Isometric core endurance

In this study, we suggested similar endurance strength gains from LL-BFRT with a lower load compared to HL-ST. It is widely recognized that resistance training (70%–85% of 1RM) is effective for rapidly enhancing muscle strength and endurance, even improving neural adaptations (7). However, previous studies indicate that low-load resistance training with BFR increases muscle strength and endurance (10–12). Improvements observed in our work are consistent with these results, with LL-BFRT displaying increased strength endurance. This effect may be attributed to the hypoxic and ischemic environment caused by BFR. When muscles contract under pressure, blood flow to the limbs is restricted, reducing arterial inflow and limiting venous return. It leads to localized hypoxia and the accumulation of metabolic byproducts (9). The recruitment of type I muscle fibers is decreased in the hypoxic environment of the muscle, but type II muscle fibers, which rely on anaerobic metabolism, are more easily recruited and activated (27). Previous research indicates that LL-BFRT can effectively stimulate type II muscle fibers, similar to HL-RT (28). Consequently, there is a similar muscle fiber activation effect between HL-RT and LL-BFRT. Furthermore, the BFR stimulus is more effective at promoting the release of anabolic hormones that are beneficial for muscle growth than resistance training. High levels of metabolic stress are generated during the LL-BFRT (9, 11). LL-BFR increases venous pooling and metabolic load, leading to a buildup of byproducts that raise lactate levels (29). Metabolic reactions are enhanced in the low-pH environment caused by lactate accumulation, stimulating anabolic hormones that boost muscle protein synthesis (9, 11). The growth hormone IGF-1 and testosterone in young men increased effectively after LL-BFRT (30). Satoshi Fujita et al. observe that BFR training boosts the activity of protein synthesis enzymes, like S6K1, and significantly improves muscle protein synthesis within 3 h post-training (31). When performing high-load exercises, athletes' technical flaws or poor postures place improper mechanical stress on muscles, leading to lumbar muscle atrophy and strength imbalance, which is the main cause of low back pain in athletes (32). Muscle strength gains play a crucial role in enhancing endurance (33). Thus, isometric core endurance improvement in the LL-BFRT group is associated with training under BFR, which stimulates neuromuscular activity and secretes the anabolic hormones, thereby recruiting more muscle fibers.

Regarding isometric core flexor endurance, neither group improved significantly. Patients with CNLBP often have weakened trunk extensor strength, while flexor strength remains similar to healthy individuals (34). It may explain why flexor muscles showed no difference in this study.

The cuffs were placed on the limbs, but we observed a significant increase in core strength, likely attributable to BFRT-derived remote muscle strength adaptations. May et al. propose that, with the same training intensity and load, the lower limbs with BFR significantly enhanced upper limb strength, while no such effect was observed in the non-BFR group (35). LL-BFRT of bench-press increases both triceps and pectoralis size and strength, even when only restricted to the triceps, as observed by Yasuda et al. (36). Although BFR is limited to the limbs, it may indirectly enhance the training effect of unrestricted muscles (36, 37). After BFRT, a remote strength transfer is noticed, enhancing the strength of the core muscles that are trained simultaneously. This transfer effect may be linked to neural interactions between muscles, elevated growth factor levels, and overall adaptive responses (9, 29). BFRT could generate anabolic hormones and proteins that promote muscle growth and circulate throughout the body (30). Importantly, BFRT-derived remote strength transfer occurs only in muscles stimulated during exercise simultaneously (38). The underlying mechanism of remote strength transfer in BFRT still requires further investigation.

### 4.2 Pain intensity and functional disability level

VAS and ODI for low back pain are among the most commonly used tools for lumbar spine surgery patients (16). We found encouraging results in the LL-BFRT group: significant improvements were observed in pain intensity and functional disability level. Previous studies have found that pain, joint swelling, and joint mobility can be significantly improved through LL-BFRT after knee surgery (39). The reason may be that LL-BFRT effectively induces endogenous analgesic mechanisms and the opioid system (40). Group III and IV afferent fibers are activated by BFR to transmit pain signals, which stimulates opioid receptors in the central nervous system. Rodrigues et al. further underscore that the tenderness threshold in the limbs remained elevated above baseline for 24 h after BFRT, suggesting that BFRT provides long-term benefits for pain relief (40). The safety of BFR is particularly noteworthy because heavy loads exert pressure on the joints, leading to exercise-related pain, fatigue, or intolerance (9). Nevertheless, LL-BFRT, which achieves similar training effects to HL-RT with a lower load, could reduce mechanical stress on the musculature and the risk of injury during training (39). Patients with knee osteoarthritis are observed to have aggravated swelling, inflammation, and pain after HL-RT (41). For athletes with chronic pain, repetitive weightlifting further damages already injured and painful joints (42). Six weeks of LL-BFRT can improve function and reduce pain in patients with lateral elbow tendinopathy (19).

The HL-RT group showed less improvement in pain intensity and functional disability level than the LL-BFRT group, while the VAS and ODI also decreased in HL-RT. The reflex inhibition caused by low back pain leads to varying degrees of disuse atrophy in the muscles, reinforcing the vicious cycle of pain and muscle atrophy. HL-RT restores proper biomechanical patterns of the lumbar spine and reduces pain due to enhanced core endurance.

### 4.3 Isokinetic core strength

The LL-BFRT group and the HL-RT group showed significant differences in isokinetic core extensors PT/BW at 120°/s and 30°/s, respectively. In contrast, our study neither found any statistically significant isokinetic strength gains in the core flexors in the groups. Peak torque at high-speed reflects the ability to generate force rapidly (43). At 120°/s, extensors PT/BW was increased significantly, likely because the hypoxic environment created by BFRT is more effective for recruiting type II muscle fibers, which are responsible for faster force generation (28). However, HL-RT showed more remarkable improvement in low-speed isokinetic movements assessing maximal torque values. Heavy-load training has a more significant impact by stimulating neuromuscular adaptations. F/E is the ratio of the trunk flexors to extensor's peak torque in the isokinetic strength test, reflecting the trunk stability and muscle strength balance. Patients with low back pain experience varying degrees of strength decline in flexor and extensor muscles. Nevertheless, the decrease in extensor strength is more pronounced than that in flexor strength. This leads to a higher F/E value than in healthy individuals (44). After the intervention, both training programs resulted in a decline in F/E, indicating that LL-BFRT and HL-RT improved coordination of the lumbar in athletes with CNLBP. LL-BFRT significantly improved at 120°/s and is more effective than HL-RT in enhancing the balance of trunk muscles during rapid movements.

## 5 Limitations

There are some limitations associated with the present study. Due to time limitations, this study conducted 4 weeks of training. More evidence will be provided if the intervention is carried out over 8–12 weeks. In addition, if we recorded the outcomes after each exercise session, the effectiveness of BFRT could be explored in more depth.

## 6 Conclusion

This is the first study on LL-BFRT in CNLBP and enters a new research field in lumbar rehabilitation. We concluded herein that LL-BFRT significantly reduced pain and improved functional disability compared to HL-RT. LL-BFRT was equally effective in improving core strength as HL-RT at a lower load in male collegiate athletes with CNLBP. Therefore, the LL-BFRT program used in this study may be applied to training patients with CNLBP, especially athletes who need to improve muscle strength and isometric endurance. Future studies may investigate how BFR can be integrated with diverse physical training as rehabilitation programs for populations with localized movement disorders.

## Data Availability

The raw data supporting the conclusions of this article will be made available by the authors, without undue reservation.
